# MethylRAD Sequencing Technology Reveals DNA Methylation Characteristics of *Apostichopus japonicus* of Different Ages

**DOI:** 10.3390/ani13223530

**Published:** 2023-11-15

**Authors:** Xinyu Yang, Lingshu Han, Qi Ye, Hao Wang, Jinyuan Zhang, Wenpei Wang, Haoran Xiao, Yongjie Wang, Luo Wang, Jun Ding

**Affiliations:** 1 Liaoning Provincial Key Laboratory of Northern Aquatic Germplasm Resources and Genetics and Breeding, Dalian Ocean University, Dalian 116023, China; 2 Key Laboratory of Mariculture & Stock Enhancement in North China’s Sea, Ministry of Agriculture and Rural Affairs, Dalian Ocean University, Dalian 116023, China; 3 School of Marine Sciences, Ningbo University, Ningbo 315832, China

**Keywords:** MethylRAD-Seq, *Apostichopus japonicus*, body wall, age identification

## Abstract

**Simple Summary:**

In this study, MethylRAD-Seq of the body wall tissues of *Apostichopus japonicus* at different ages was analyzed based on methylated RAD-Seq technology and, combined with GO and KEGG analyses, different genes related to the age of *A. japonicus* were screened, such as *H2AX*, *Hsp90*, *Pepn*, and *CDC6*. This provides reference significance for the identification of *A. japonicus* age.

**Abstract:**

The *A. japonicus* industry has expanded significantly, but no research has focused on determining the age of *A. japonicus* during farming. Correctly estimating the age of *A. japonicus* can provide a decision-making basis for the breeding process and data for the protection of *A. japonicus* aquatic germplasm resources. DNA methylation levels in the body wall of Apostichopus japonicus at 4 months, 1 year, 2 years, and 3 years old were determined using MethylRAD-Seq, and differentially methylated genes were screened. A total of 441 and 966 differentially methylated genes were detected at the CCGG and CCWGG sites, respectively. Aspartate aminotransferase, succinate semialdehyde dehydrogenase, isocitrate dehydrogenase, the histone *H2AX*, heat shock protein *Hsp90*, aminopeptidase N, cell division cycle *CDC6*, Ras GTPase activating protein (*RasGAP*), slit guidance ligand *slit1*, integrin-linked kinase *ILK*, mechanistic target of rapamycin kinase *Mtor*, protein kinase A *Pka*, and autophagy-related 3 *atg3* genes may play key roles in the growth and aging process of *A. japonicus*. This study provides valuable information regarding age-related genes for future research, and these candidate genes can be used to create an “epigenetic clock”.

## 1. Introduction

*Apostichopus japonicus*, which belongs to the *Stichopodidae* family (class Holothuroidea; phylum Echinodermata), has significant economic and medicinal value [1]. Data from the Fisheries Administration of the Ministry of Agriculture and Rural Affairs show that the total production of *A. japonicus* in China increased from 197,000 tonnes in 2020 to 223,000 tonnes in 2021 [2]. The *A. japonicus* industry in China has expanded significantly, but no research has focused on determining the age of *A. japonicus* during farming. Correctly estimating the age of *A. japonicus* can provide a decision-making basis for the breeding process, assist in identifying developmental stages and breeding time, evaluate population resources, analyze population dynamics, and provide data for the protection of *A. japonicus* aquatic germplasm resources.

The age and variety of *A. japonicus* can often be determined by observing the appearance, gonad development, and bone fragments of specimens. Zhang et al. examined the varieties and shapes of bone fragments in *A. japonicus* at different ages and discovered that the proportion of bone fragments in the same tissue of *A. japonicus* was different at different ages [3]. Wang et al. observed the table shape of *A. japonicus* at ages and discovered that the number of table holes in the chassis tended to decrease with age, and the outer margin of the table shape degenerated from uneven to smooth [4].

Venney et al. demonstrated that, in *Oncorhynchus tshawytscha*, changes in DNA methylation were associated with genes, tissues, and age and showed that decreases in DNA methylation with age and tissue-specific methylation patterns varied [5]. Anastasiadi et al. amplified 48 CpG sites from four genes in muscle samples of Dicentrarchus labrax, for which the age was accurately determined by targeting the sodium bisulfite sequences. They then applied penalized regression to predict age and constructed an epigenetic clock in fish [6]. Methylation is a significant epigenetic modification of eukaryotic genomic DNA, and it plays crucial roles in biological processes such as gene expression, embryonic development, cell differentiation, and gene imprinting [7,8]. High-throughput sequencing has been widely used in studies of aquatic animal development [9], disease [10], and age [11]. Using an optimized unique set of 26 CpG sites and a *Daniorerio* age prediction model based on DNA methylation, Mcgaughey et al. developed a multiplex PCR assay with a mean median absolute error rate of 3.2 weeks for age prediction [12]. Montesanto et al. determined an epigenetic clock for Chelonia mydas with a median absolute error of 2.1 years based on DNA methylation levels at 18 CpG sites [13]. De Paoli-Iseppi et al. used DNA methylation biomarkers to estimate the age of Ardenna tenuirostris and identified seven CpG sites where DNA methylation levels correlated with age. Ages estimated using models constructed based on these correlations had an average difference of 2.8 years from known ages [14]. Although age prediction based on DNA methylation levels is a swiftly developing field of epigenetics, no studies using DNA methylation levels to determine the age of *A. japonicus* have been reported so far.

To gain a deeper understanding of the differences between the DNA methylation levels of *A. japonicus* at different ages, we used MethylRAD-Seq to screen and identify differentially methylated genes and used Gene Ontology (GO) and Kyoto Encyclopedia of Genes and Genomes (KEGG) enrichment analyses to determine the functional relationships between these genes. Our results provide a theoretical foundation for biological research and age determination of *A. japonicus*.

## 2. Materials and Methods

### 2.1. Experimental Materials

Healthy 3-year-old (weight: 175.59 ± 34.38 g) (A), 2-year-old (weight: 42.51 ± 19.33 g) (B), 1-year-old (body weight: 5.19 ± 6.26 g) (C), and 4-month-old (body weight: 1.72 ± 1.13 g) (D) *A. japonicus* were cultivated at a key laboratory in March 2019, March 2020, March 2021, and December 2021. Specimens were grown in the same place: a 600 L aquaculture pond with water at 14 ± 1.5 °C, a salinity of 30 ± 1, and a pH of 7.0. The experiment began in March 2022. Six *A. japonicus* were picked randomly from each group, the body wall was removed, and the *A. japonicus* were snap-frozen in liquid nitrogen and maintained at −80 °C.

### 2.2. Reagent Preparation

The synthesized primers and linker dry powder were centrifuged at high speed at room temperature, suspended, and mixed with water to obtain a storage solution at a concentration of 100 µM. A linker working solution was prepared at a concentration of 5 µM and a primer was prepared at a concentration of 10 µM before the experiment, and the working fluids were stored at −20 °C for later use. Then, 10% (wt/vol) APS (St. Louis, MO, USA. Sigma-Aldrich, cat. no. A3678) was added to 1 g of APS and dissolved in a small amount of water for bandwidth evaluation to 10 mL. The solution was stored at −20 °C for 3–6 months. Then, 1% (wt/vol) agarose gel, weighing 0.4 g, was dissolved in 40 mL 1× TAE buffer (Sigma-Aldrich, cat. no. T8280), heated until the agarose was prepared, and then we waited for the solution to cool down to about 60 °C, after which 4 µL of SYBR Safe DNA dye was added, shaken well, and poured in a glue tank at room temperature, which was used after the glue solidified. The agarose glue was used to prepare an 8% (wt/vol) polyacrylamide gel. A total of 5.4 mL of acrylamide (29:1), 4 mL of 5× TBE (Sigma-Aldrich, cat. No. T3913), 280 µL of 10% (wt/vol) APS, 10 µL of TEMED, and 10.6 mL of pure water were mixed together by shaking, poured into a pre-clamped rubber plate, and left at room temperature for at least 1 h before use, after which the 8% polyacrylamide gel could be used.

### 2.3. MethylRAD Experimental Process

(1) We implemented the enzyme digestion reaction system as shown in Table 1: a control group was set, and the reaction was performed at 37 °C for 4 h;

(2) An amount of 5 µL of each control group and enzyme digestion group was detected by 1% (wt/vol) agarose gel electrophoresis and 100 V electrophoresis for 10–15 min. The effect of digestion was observed under ultraviolet light;

(3) We then implemented the linking reaction system as shown in Table 2: the reaction conditions were a 4 °C connection for 6–8 h;

(4) We then implemented the PCR reaction system and conditions as shown in Table 3;

(5) A total of 20 µL of PCR product and 1 µL of 100-bp DNA ladder were examined by electrophoresis with 8% polyacrylamide gel at 400 V for 35 min;

(6) After electrophoresis, SYBR Safe DNA dye was used for 3 min to observe the brightness of the target band (100 bp);

(7) We then cut the desired strip and placed it into a 1.5 mL centrifuge tube, ground the glue with a grinding rod, added 30–40 µL of pure water, and let it stand at 4 °C 6–12 h;

(8) We then introduced the barcode sequence and the PCR reaction system as shown in Table 4;

(9) We then purified PCR products with the QIAquick PCR Purification Kit, eluted them with 15 µL of pure water, and then used QubitQuantity for determination. In general, the ideal concentration of purified products is 10–30 ng/µL;

(10) If multiple libraries are built, libraries with different barcode numbers can be mixed according to the amount of sent measurement data. The combined library concentration is more suitable at 5–10 ng/µL;

(11) The mixed libraries were sequenced using the Illumina Novaseq PE150 sequencing platform. Primer and linker sequences are shown in Table 5.

### 2.4. Data Analysis Process

(1) We first checked the raw data obtained from sequencing for quality. If there were more than 15% low-quality bases or sequences with too many N bases in the acquired reads, they were removed;

(2) We then aligned enzyme reads to the reference genome using bowtie2 (version 2.3.4.1) software (parameter settings: −M = 4, −v = 2, −r = 0) to identify reliable methylation sites;

(3) Then, we utilized SNPeff software (version: 4.1) to extract the UTR region based on annotation information followed by Bedtools software (version: v2.18.0) to determine the distribution of methylation sites in various gene elements in the sample [15,16];

(4) Using DESeq software (version: v1.18.0), we calculated the difference *p* value and difference multiple (Log2FC) of each site between the groups, combined the sequencing depth of each site in each sample, and compared the methylation levels between the two groups;

(5) We then screened the genes for which the difference between groups was *p ≤* 0.05 and |log2FC| > 1 and organized their methylation level and annotation data;

(6) Finally, we conducted GO and KEGG enrichment analyses of differential genes

## 3. Results

### 3.1. MethylRAD-Seq Data and Identification of A. japonicus DNA Methylation Sites

We performed MethylRAD-Seq of genomic DNA extracted from the body wall tissue of *A. japonicus* at 4 months, 1 year, 2 years, and 3 years old. We used a total of 12 tissue samples (3 samples from each of the age groups) and obtained a total of 280,422,936 reads; a total of 130,276,536 (46.4%) of them were high-quality reads. The three samples of 3-year-old *A. japonicus* yielded 75,848,311 reads; a total of 36,771,351 (48.48%) of them were high-quality reads, with an average of 25,282,770. The three samples of 2-year-old *A. japonicus* yielded 62,579,986 reads; a total of 32,326,130 (51.66%) of them were high-quality reads, with an average of 20,859,995 The three samples of 1-year-old *A. japonicus* yielded 65,336,130 reads; a total of 30,392,328 (46.52%) of them were high-quality reads, with an average of 21,778,710. The three samples of 4-month-old *A. japonicus* yielded 76,656,509 reads; a total of 30,786,727 (40.16%) of them were high-quality reads, with an average of 25,552,170. See Table 6 and Table 7 for details. Identification and analysis of methylation sites of the enzyme-produced DNA fragments from the 12 tissue samples showed that the proportion of CCGG-type methylation sites was substantially higher than that of CCWGG-type methylation sites. The average numbers of CCGG methylation sites for the 3-, 2-, and 1-year- and 4-month-old.

*A. japonicus* were 70,357, 80,376, 79,065, and 82,131, respectively. The average numbers of CCWGG methylation sites for the 3-, 2-, and 1-year- and 4-month-old *A. japonicus* were 7755, 11,379, 11,524, and 11,833, respectively (Table 8).

### 3.2. Identification of Differentially Methylated Genes

**Figure 1:**
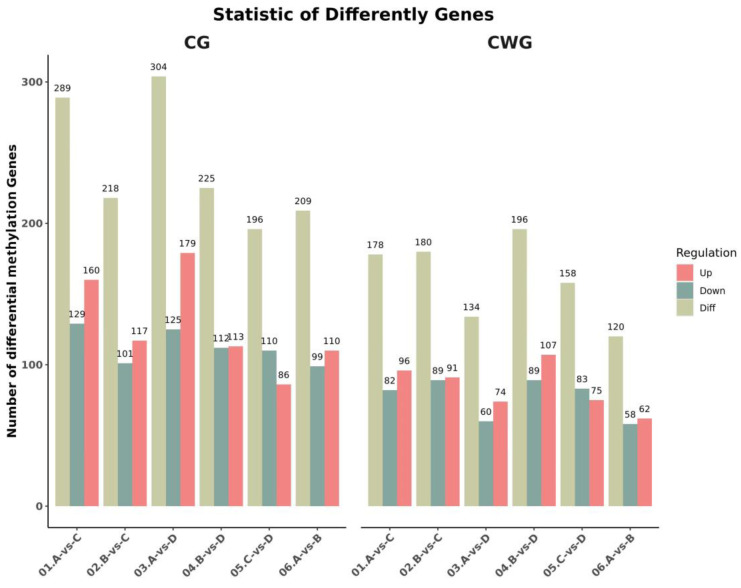
Statistical histogram of differentially methylated genes between groups (3-year-old (A), 2-year-old (B), 1-year-old (C), 4-month-old (D). 01: 3-year-old vs. 1-year-old; 02: 2-year-old vs. 1-year-old; 03: 3-year-old vs. 4-month-old; 04: 2-year-old vs. 4-month-old; 05: 1-year-old vs. 4-month-old; 06: 3-year-old vs. 2-year-old).

Genes were considered to be differentially methylated between the age groups when the *p*-value was ≤0.05 and the absolute value of the log2fold change was >1. In the 3-year vs. 2-year comparison, we identified 209 (99 up-regulated, 110 down-regulated) and 120 (62 up-regulated, 58 down-regulated) differentially methylated genes at the CCGG and CCWGG sites, respectively. In the 3-year vs. 1-year comparison, we identified 289 (160 up-regulated, 129 down-regulated) and 178 (96 up-regulated, 82 down-regulated) differentially methylated genes at the CCGG and CCWGG sites, respectively. In the 3-year vs. 4-month comparison, we identified 304 (179 up-regulated, 125 down-regulated) and 134 (74 up-regulated, 60 down-regulated) differentially methylated genes at the CCGG and CCWGG sites, respectively. In the 2-year vs. 1-year comparison, we identified 218 (117 up-regulated, 101 down-regulated) and 180 (91 up-regulated, 89 down-regulated) differentially methylated genes at the CCGG and CCWGG sites, respectively. In the 2-year vs. 4-month comparison, we identified 225 (113 up-regulated, 112 down-regulated) and 196 (107 up-regulated, 89 down-regulated) differentially methylated genes at the CCGG and CCWGG sites, respectively. In the 1-year vs. 4-month comparison, we identified 196 (86 up-regulated, 110 down-regulated) and 158 (75 up-regulated, 83 down-regulated) differentially methylated genes at the CCGG and CCWGG sites, respectively. The specific quantities of and changes in these comparisons are shown in Figure 1. At the CCGG sites, the highest number of differentially methylated genes (304) was found in the 3-year vs. 4-month comparison, and the lowest number (196) was found in the 1-year vs. 4-month comparison. At the CCWGG sites, the highest number of differentially methylated genes (196) was found in the 2-year vs. 4-month comparison, and the lowest number (120) was found in the 3-year vs. 2-year comparison.

### 3.3. GO Enrichment Analysis of Differentially Methylated Genes

**Figure 2:**
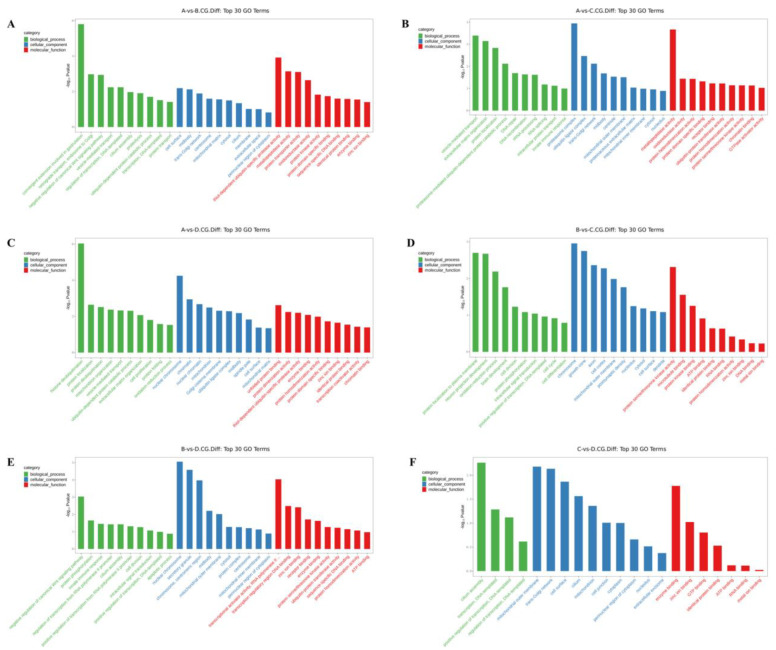
Histogram of differentially methylated genes’ GO functional classification: CCGG. (**A**) A vs. B; (**B**) A vs. C; (**C**) A vs. D; (**D**) B vs. C; (**E**) B vs. D; (**F**) C vs. D. (3-year-old (A), 2-year-old (B), 1-year-old (C), 4-month-old (D)).

**Figure 3:**
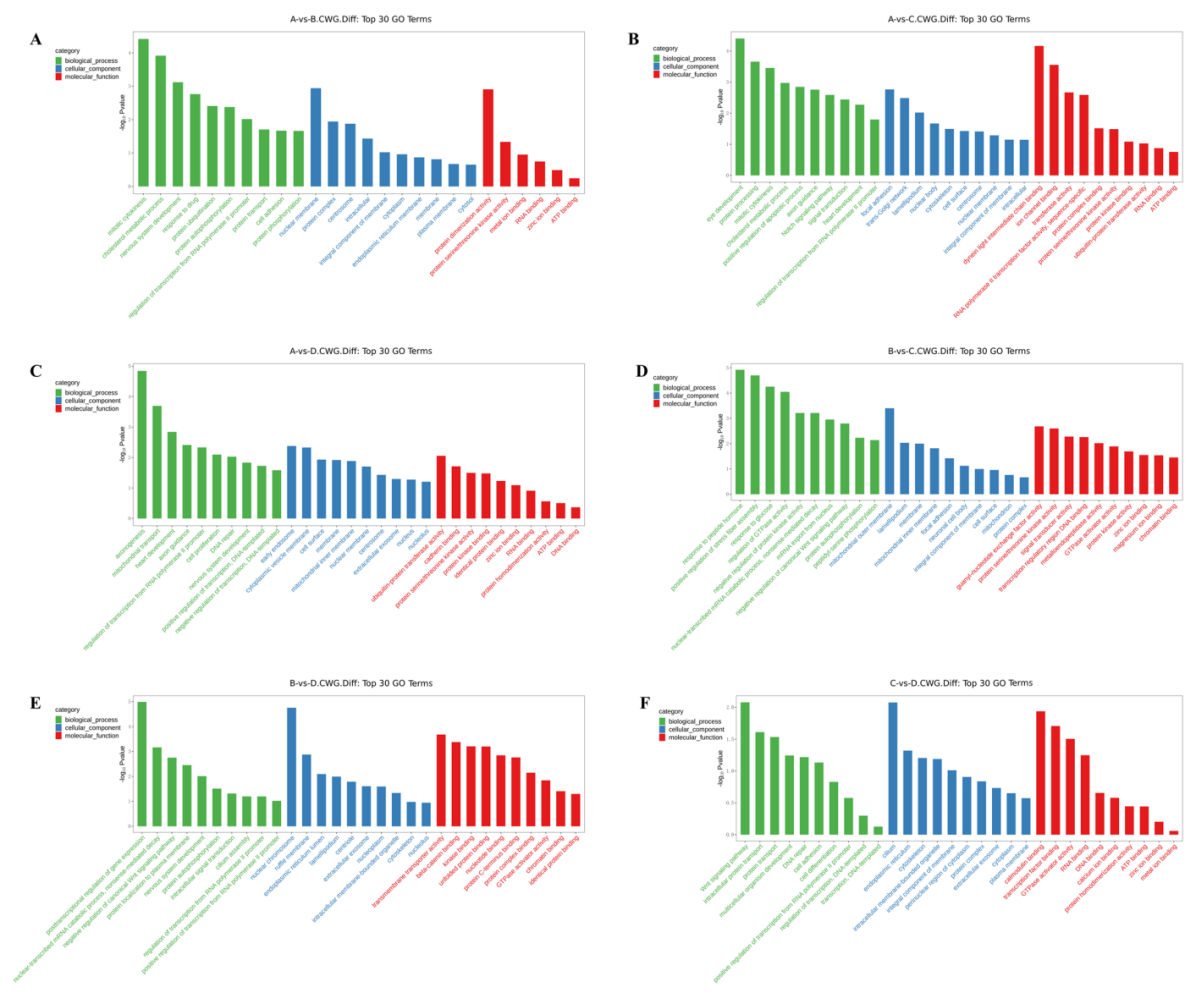
Histogram of differentially methylated genes’ GO functional classification: CCWGG. (**A**) A vs. B; (**B**) A vs. C; (**C**) A vs. D; (**D**) B vs. C; (**E**) B vs. D; (**F**) C vs. D. (3-year-old (A), 2-year-old (B), 1-year-old (C), 4-month-old (D)).

The differentially methylated genes identified in each of the comparisons were annotated with GO terms under three main categories, namely biological process (BP), cellular component (CC), and molecular function (MF). The top ten enriched terms under each of the categories are displayed as bar charts (Figure 2 and Figure 3).

In the 3-year vs. 2-year comparison, the differentially methylated genes at CCGG sites were significantly enriched in the BP terms convergence extension and retrograde transport of gastrulation, endosome to Golgi, and negative regulation of typical Wnt signaling pathways; the CC terms cell surface, midsole, and trans-Golgi network; and the MF terms thiol-dependent ubiquitin-specific protease activity, metalloproteinase activity, and protein transporter activity. The differentially methylated genes at CCWGG sites were significantly enriched in the BP terms mitotic cell division, cholesterol metabolism, and nervous system development; the CC terms nuclear membranes, protein complexes, and centrosomes; and the MF terms protein dimerization activity, protein serine/threonine kinase activity, and metal ion binding. The results are shown in Figure 2A and Figure 3A.

In the 3-year vs. 1-year comparison, the differentially methylated genes at CCGG sites were significantly enriched in the BP terms vesicle-mediated transport, extracellular matrix organization, and protein localization; the CC terms proteasome complex, ubiquitin ligase complex, and trans-Golgi network; and the MF terms metalloproteinase activity, oxidoreductase activity, and protein heterodimerization activity. The differentially methylated genes at CCWGG sites were significantly enriched in the BP terms eye development, protein processing, and mitotic cell division; the CC terms adhesion, trans-Golgi network, and plate-like pods; and the MF terms dynein light intermediate chain binding, ion channel binding, and transferase activity. The results are shown in Figure 2B and Figure 3B.

In the 3-year vs. 4-month comparison, the differentially methylated genes at CCGG sites were significantly enriched in the BP terms histone deubiquitination, protein localization, and protein deubiquitination; the CC terms nuclear chromosomes, chromatin, and nuclear chromatin; and the MF terms unfolded protein binding, protein dimerization activity, and thiol-dependent ubiquitin-specific protease activity. The differentially methylated genes at CCWGG sites were significantly enriched in the BP terms axonogenesis, mitochondrial transport, and cardiac development; the CC terms early endosomes, cytoplasmic vesicle membranes, and cell surfaces; and the MF terms ubiquitin-protein transferase activity, cadherin binding, and protein serine/threonine kinase activity. The results are shown in Figure 2C and Figure 3C.

In the 2-year vs. 1-year comparison, the differentially methylated genes at CCGG sites were significantly enriched in the BP terms protein localization to plasma membrane, nerve cell projection development, and redox process; the CC terms chromosomes, growth cones, and axons; and the MF terms protein serine/threonine kinase activity, microtubule binding, and protein kinase binding. The differentially methylated genes at CCWGG sites were significantly enriched in the BP terms response to peptide hormones, forward regulation of stress fiber assembly, and response to glucose; the CC terms outer mitochondrial membrane, plate-shaped pods, and membranes; and the MF terms ornithine-nucleotide exchange factor activity, protein serine/threonine kinase activity, and signal sensor activity. The results are shown in Figure 2D and Figure 3D.

In the 2-year vs. 4-month comparison, the differentially methylated genes at CCGG sites were significantly enriched in the BP terms negative regulation of typical Wnt signaling pathways, protein phosphorylation, and innate immune response; the CC terms nuclear chromosomes, secretory granules, chromosomes, and centromere regions; and the MF terms transcriptional activation activity, RNA polymerase II, transcriptional regulatory region sequence-specific DNA binding, transcription regulatory region DNA junction, and zinc ion binding. The differentially methylated genes at CCWGG sites were significantly enriched in the BP terms post-transcriptional regulation of gene expression, nuclear transcription mRNA catabolism, senseless-mediated decay, and negative regulation of the typical Wnt signaling pathway; the CC terms nuclear chromosomes, ruffle membranes, and endoplasmic reticulum cavity; and the MF terms transmembrane transporter activity, β-catenin binding, and kinase binding. The results are shown in Figure 2E and Figure 3E.

In the 1-year vs. 4-month comparison, the differentially methylated genes at CCGG sites were significantly enriched in the BP terms cilia components, transcription, DNA templating, forward regulation of transcription, and DNA templating; the CC terms mitochondrial outer membrane, trans-Golgi network, and cell surface; and the MF terms enzyme binding, zinc ion binding, and GTP binding. The differentially methylated genes at CCWGG sites were significantly enriched in the BP terms ciliary components, transcription, DNA templating, forward regulation of transcription, and DNA templating; the CC terms cilia, endoplasmic reticulum, and cytoskeleton; and the MF terms calmodulin binding, transcription factor binding, and GTPase activator activity.

These results show that differentially methylated genes were involved in various essential biological processes, including negative regulation of the typical Wnt signaling pathway, protein localization, and mitotic cell division, as well as with various cellular components, including the trans-Golgi network, centrosome, nuclear chromosome, mitochondrial outer membrane, cell surface, and plate viburnum. The differentially methylated genes were also involved in multiple molecular functions, including thiol-dependent ubiquitin-specific protease activity, metalloprotease activity, protein dimerization activity, protein serine/threonine kinase activity, and zinc ion binding. The results are shown in Figure 2F and Figure 3F.

### 3.4. KEGG Enrichment Analysis of Differentially Methylated Genes

The differentially methylated genes identified in each of the comparisons were annotated with KEGG pathways.

**Figure 4:**
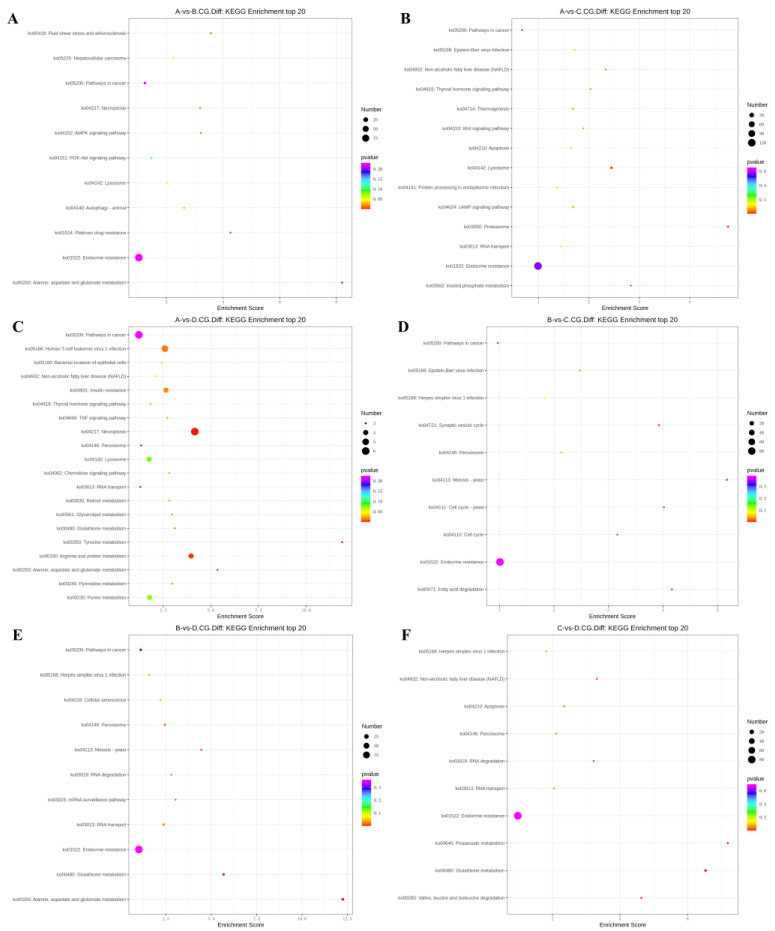
KEGG top 20 bubble chart of differentially methylated genes at CCGG sites. (**A**) A vs. B; (**B**) A vs. C; (**C**) A vs. D; (**D**) B vs. C; (**E**) B vs. D; (**F**) C vs. D. (3-year-old (A), 2-year-old (B), 1-year-old (C), 4-month-old (D)).

**Figure 5:**
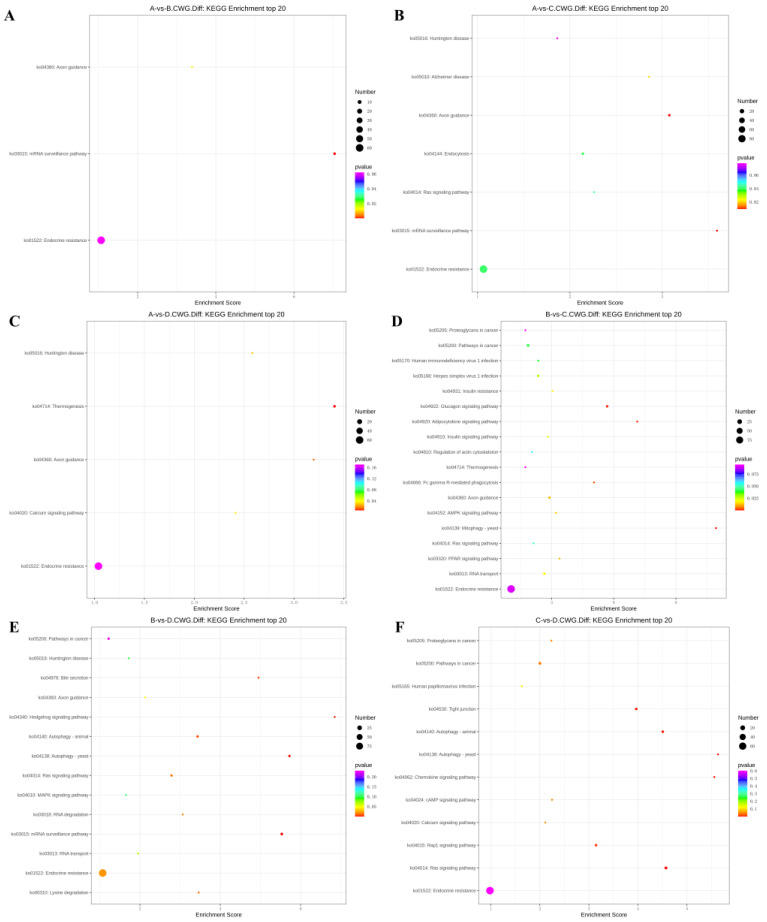
KEGG top 20 bubble chart of differentially methylated genes at CCWGG sites. (**A**) A vs. B; (**B**) A vs. C; (**C**) A vs. D; (**D**) B vs. C; (**E**) B vs. D; (**F**) C vs. D. (3-year-old (A), 2-year-old (B), 1-year-old (C), 4-month-old (D)).

In the 3-year vs. 2-year comparison, the differentially methylated genes at CCGG sites were significantly enriched in alanine, aspartate, and glutamic acid metabolism, platinum resistance, fluid shear stress, and atherosclerosis; up-regulated genes were significantly enriched in alanine, aspartate, and glutamic acid metabolism and endocrine resistance pathways, whereas down-regulated genes were significantly enriched in the AMPK signaling pathway, liver cancer, and endocrine resistance. In the alanine, aspartate, and glutamic acid metabolic pathway, aspartate aminotransferase and δ-1-pyrroline-5-carboxylate dehydrogenase were up-regulated. The differentially methylated genes at CCWGG sites were significantly enriched in mRNA monitoring, axon guidance, and endocrine resistance; up-regulated genes were significantly enriched in the mRNA monitoring pathway and endocrine resistance, whereas down-regulated genes were significantly enriched in endocrine resistance pathways (Figure 3). In the mRNA monitoring pathway, *FIP1*, *SYMPK*, and *smg1* were up-regulated, and *smg6* was down-regulated. In the axon guidance pathway, the Ras GTPase activating protein gene *rasgap* and *limk* were down-regulated, and slit guidance ligand *slit1* was up-regulated. The results are shown in Figure 4A and Figure 5A.

In the 3-year vs. 1-year comparison, the differentially methylated genes at CCGG sites were significantly enriched in proteasome, lysosome, and inositol phosphate metabolism; up-regulated genes were significantly enriched in lysosome, thermogenesis, and endocrine resistance, whereas down-regulated genes were significantly enriched in inositol phosphate metabolism and endocrine resistance. In the lysosomal pathway, histone, *SGSH*, and *GNPT* were up-regulated, and *MPR* and *AP.4* were down-regulated. In the inositol phosphate metabolism pathway, phosphatidylinositol 4,5-diphosphate 3-kinase catalyzes the down-regulation of subunit β isoform and inositol-3-phosphate synthase 1-A. The differentially methylated genes at CCWGG sites were significantly enriched in axon guidance, mRNA monitoring, and Alzheimer’s disease; up-regulated genes were significantly enriched in mRNA monitoring pathways and endocrine resistance, whereas down-regulated genes were significantly enriched in Alzheimer’s disease, endocytosis, and endocrine resistance. In the mRNA monitoring pathway, *FIP1*, *SYMPK*, and *smg1* were up-regulated. In the axon guidance pathway, *rasgap* and integrin-linked kinase *ILK* were down-regulated, and *slit1* and *slit2* were up-regulated. In the Alzheimer’s disease pathway, *ncstn*, *aphl-1*, and *cxv* were down-regulated. In the endocrine resistance pathway, *ILK* was down-regulated. The results are shown in Figure 4B and Figure 5B.

In the 3-year vs. 4-month comparison, the differentially methylated genes at CCGG sites were significantly enriched in tyrosine metabolism, necrotizing apoptosis, and alanine, aspartic acid, and glutamic acid metabolism; up-regulated genes were significantly enriched in necrotizing apoptosis, alanine, aspartic acid, and glutamic acid metabolism, and human T-cell leukemia virus 1 infection, whereas down-regulated genes were significantly enriched in glutathione metabolism, non-alcoholic fatty liver disease, and purine metabolism. In the alanine, aspartic acid, and glutamic acid metabolism pathway, the genes encoding aspartate aminotransferase, succinate semialdehyde dehydrogenase, and amide phosphate ribosyltransferase were up-regulated. In the necrotizing apoptosis pathway, *ANT*, *PYGL*, heat shock protein *Hsp90*, *DRP1*, and *ESCRT-III* were up-regulated, and the histone *H2AX* was down-regulated. In the glutathione metabolic pathway, the aminopeptidase N (*pepn*) and the isocitrate dehydrogenase genes were down-regulated. The differentially methylated genes at CCWGG sites were significantly enriched in thermogenesis, axon guidance, and Huntington’s disease. The most enriched pathway was the secretory resistance pathway with a total of 62 differentially methylated genes; among them, up-regulated genes were significantly enriched in endocrine resistance pathways, and down-regulated genes were significantly enriched in endocrine resistance pathways. In the endocrine resistance pathway, the protein kinase gene *Pka* was down-regulated. In the axonal guidance pathway, *RGS3* and *ROBO1* were down-regulated and *slit1* was up-regulated. The results are shown in Figure 4C and Figure 5C.

In the 2-year vs. 1-year comparison, the differentially methylated genes at CCGG sites were significantly enriched in meiosis—yeast, fatty acid degradation, and cell cycle—yeast; up-regulated genes were significantly enriched in Epstein–Barr virus infection, peroxisome, and endocrine resistance, whereas down-regulated genes were significantly enriched in meiosis—yeast, cell cycle, and endocrine resistance. In the meiosis—yeast pathway, *GLC7*, *IN1*, and cell division control *CDC6* were down-regulated. The differentially methylated genes at CCWGG sites were significantly enriched in mitochondrial autophagy—yeast, the glucagon signaling pathway, and the adipocytokine signaling pathway. The most enriched pathway was the endocrine resistance pathway with a total of 92 differentially methylated genes; among them, up-regulated genes were significantly enriched in the glucagon signaling pathway, axon guidance, and cancer pathways, whereas down-regulated genes were significantly enriched in endocrine resistance and cancer pathways. In the axon guidance pathway, *limk* and *ptch1* were up-regulated. In the endocrine resistance pathway, the mechanistic target of rapamycin kinase gene *mtor* and *ILK* was down-regulated. In the glucagon signaling pathway, *sik2*, *cpt1*, and *phk* were up-regulated, and *gys* was down-regulated. The results are shown in Figure 4D and Figure 5D.

In the 2-year vs. 4-month comparison, the differentially methylated genes at CCGG sites were significantly enriched in alanine, aspartate, and glutamic acid metabolism, glutathione metabolism, and meiosis—yeast; up-regulated genes were significantly enriched in alanine, and endocrine resistance, whereas down-regulated genes were significantly enriched in glutathione metabolism, meiosis—yeast, and ribonucleic acid transport. In alanine, aspartate, and glutamic acid metabolic pathways, the genes encoding aspartame synthase and hypothetical protein BSL78_23121 were down-regulated, and the genes encoding isoaspartic acid peptidase/L-asparaginase-like, succinate semialdehyde dehydrogenase, and 4-aminobutyrate aminotransferase were up-regulated. *Pepn* and the isocitrate dehydrogenase gene were down-regulated in the glutathione metabolic pathway. *GLC7*, *IN1*, and *CDC6* were down-regulated in the meiosis—yeast pathway. The differentially methylated genes at CCWGG sites were significantly enriched in mRNA monitoring pathways, autophagy—yeast, and hedgehog signaling pathways. The most enriched pathway was the endocrine resistance pathway with a total of 96 differentially methylated genes; among them, up-regulated genes were significantly enriched in RNA degradation, axon guidance, and Ras signaling pathways, whereas down-regulated genes were significantly enriched in autophagy—yeast, autophagy—animal, and mRNA monitoring pathways. In the mRNA monitoring pathway, *CPSF6/7* and *SYMPK* were down-regulated, and *smg1* and *smg6* were up-regulated. In the axonal guidance pathway, Ras GTPase activating protein *rasgap*, *limk*, and *ptch1* were up-regulated. In the autophagy—yeast pathway, *Pka*, and the autophagy-related genes *atg2* and *atg3* were down-regulated, and *e1f2α* was up-regulated. In the Ras signaling pathway, *rasgap*, *rlip76*, and *ra1bp1* were up-regulated. In the autophagy—animal pathway, *atg2*, *Pka*, *atg3*, and *e1f2α* were down-regulated. The results are shown in Figure 4E and Figure 5E.

In the 1-year vs. 4-month comparison, the differentially methylated genes at CCGG sites were significantly enriched in glutathione metabolism, propionic acid metabolism, and valine, leucine, and isoleucine degradation; up-regulated genes were significantly enriched in propionic acid metabolism and endocrine resistance, whereas down-regulated genes were significantly enriched in glutathione metabolism, apoptosis, and herpes simplex virus 1 infection. In the glutathione metabolism pathway, *pepn* and the gene encoding 6-phosphogluconate dehydrogenase were down-regulated. In the propionic acid metabolic pathway, the genes encoding malonyl-CoA decarboxylase, 4-aminobutyrate aminotransferase, and acetyl-CoA synthetase were up-regulated. The differentially methylated genes at CCWGG sites were significantly enriched in the Ras signaling pathway, autophagy—animal, and autophagy—yeast. The most enriched pathway was the endocrine resistance pathway with a total of 67 differentially methylated genes; among them, up-regulated genes were significantly enriched in cancer pathways and endocrine resistance, whereas down-regulated genes were significantly enriched in autophagy—yeast, the chemokine signaling pathway, and the autophagy—animal pathway. In the cancer pathway, *aml1*, *aml1-eto*, *aml-evi1*, *dapk*, and *ecm* were up-regulated, and *ra1* was down-regulated. In the autophagy—yeast pathway, *atg2* was down-regulated, and *e1f2α* was up-regulated. In the Ras signaling pathway, *tiam1* and *ra1* were down-regulated, and *rasgap* and *plcε* were up-regulated. In the autophagy—animal pathway, *atg2* and *e1f2α* were down-regulated, and *dapk* was up-regulated. In the chemokine signaling pathway, *Pka* was down-regulated. The results are shown in Figure 4F and Figure 5F.

## 4. Discussion

In this study, we used the MethylRAD-Seq method to identify differentially methylated genes of *A. japonicus* at four ages. By pairwise comparison and GO and KEGG functional enrichment analysis, differentially methylated genes and their regulatory trends in *A. japonicus* at the four ages were identified. See Supplementary Document S1 for details. The results provide information that can be used to determine the age of *A. japonicus*. We found that there were significantly more CCGG-type than CCWGG-type methylation sites. The CCGG methylation sites belong to the CpG type, indicating that the CCGG-type base composition is more prone to methylation. The DNA methylation of *A. japonicus* at different ages occurred mainly in gene regions. These results suggest that DNA methylation associated with the age of *A. japonicus* may play a role in regulating gene expression.

Sharma et al. (1987) studied the regulatory effect of hydrocortisone on the aspartate aminotransferase isoenzymes in rat liver and discovered that the aspartate aminotransferase content of liver increased with the age of the rats after the rats reached a certain age. [17] The up-regulation of the aspartate aminotransferase gene in *A. japonicus* may be related to the transamination in mitochondria that allows *A. japonicus* to grow and remain stable. Cathepsin b-like proteases promote aging by translocating from lysosomes to the cytoplasm and nucleus, causing oxidative stress in the cell, and contributing to the degradation of anti-aging factors [18,19]. Age-related increases in cathepsin b-like proteases may contribute to the aging of *A. japonicus.* Sun et al. discovered that the positive reactions of γ-aminobutyric acid and neurofilament protein were stronger in the retina of old cats than those in young cats, and the number of positive immune response cells increased significantly [20]. The degradation of γ-aminobutyric acid leads to the formation of succinic semialdehyde as a relatively unstable intermediate [21]. The up-regulation of the succinate semialdehyde dehydrogenase gene in *A. japonicus* may be caused by the accumulation of γ-aminobutyric acid with age, resulting in the accumulation of its degradation products. Al-Zghoul et al. studied heat stress in broiler chickens and discovered that the expression of *Hsp90* and *Hsp60* was associated with the acquisition of long-term enhanced heat tolerance [22]. Bansal et al. found that decreased intracellular *Hsp90* concentrations increased mammalian cell mortality at elevated temperatures [23]. Boehm et al. showed that basal *Hsp90* protein levels decreased with age in equine articular chondrocytes. But when *Hsp90* mRNA expression was measured in equine articular chondrocytes from post-pubertal animals, a brief and significant increase in expression was observed with the same sample size after puberty [24]. The up-regulation trend of *Hsp90* in *A. japonicus* may be attributable to oxidative stress and a disparity in sample size. Johanna et al. discovered that concentrations of the DNA damage and repair marker *γ-H2AX* decreased with age [25]. The down-regulation trend of *H2AX* in *A. japonicus* may be because of DNA damage causing gene dysregulation during aging. Miska et al. discovered that between days 9 and 20 of incubation, *Pepn* was highly expressed in *Gallus gallus*; however, its expression peaked on day 15 and decreased substantially from days 17 to 20, which is consistent with our findings in *A. japonicus* [26]. Yadav et al. discovered that NAD- and mitochondrial NADP-isocitrate dehydrogenase activity increased in male rats until adulthood and then decreased, whereas brain NADP-isocitrate dehydrogenase activity decreased progressively with age [27]. This result is consistent with the finding that, in *A. japonicus*, isocitrate dehydrogenase is most active in synthesis during development, providing 2-oxoglutarate that is then converted to glutamate and supplying a large amount of this amino acid and thereby maintaining the energy required to be active in adulthood and decreasing thereafter [27]. Markopoulos et al. discovered that the levels of proteins with cell cycle effects, such as *Cdc6*, were drastically decreased. This pattern was also observed in *A. japonicus* [28]. The activity of the lipogenic enzyme 6-phosphogluconate dehydrogenase decreased with age in adipose tissue [29], which is consistent with our findings in *A. japonicus*.

*RasGAP* proteins, which are members of the Ras family, regulate the glutamate receptor’s synaptic targets to maintain synaptic plasticity [30]. Ras GTPase is essential for the proliferation, function, and development of many cell types [31]. Srivastav et al. discovered that the expression of *RasGAP*-positive cells in mice decreased with age [32]. *RasGAP* typically increased in *A. japonicus* during adolescence and then decreased, which may be related to the decline in cell proliferation during maturity. Ethell, Morita, and Yoshida showed that multiple axon guidance molecules modulate dendritic morphology and spine growth [33,34,35]. *Slit1* may be involved in the growth of the spine of *A. japonicus*, implying that *slit1* may be associated with *A. japonicus* age on the other hand. El-Hoss et al. discovered that mouse osteoblasts cultured from primary osteoblasts deficient in *ILK* had elevated levels of cytoplasmic αNAC and increased mineralization [36]. The number of pores in bone slices of *A. japonicus* decreased with age, and *ILK* may be implicated in this process. *Mtor* (target of rapamycin) signaling influences longevity and senescence [37]. Senescence is highly regulated by the activity of TOR, an evolutionarily conserved protein kinase that regulates growth, proliferation, and cellular metabolism in response to nutrients, growth factors, and stress (Erdogan et al., 2016) [38]. With age, skeletal muscle mass naturally and gradually decreases [39]. *Mtor* may inhibit the development of skeletal muscle in *A. japonicus* by participating in the endocrine resistance pathway, thereby altering the number of bone fragments with cavities. The aging process is dependent on the effect of *PKA* down-regulation on the BKCa channels in the middle cerebral artery, which may compromise the structure and function of *PKA* [40]. Down-regulation of *PKA* in *A. japonicus* may result in senescence. In their study, Liu et al. found that *BMP9* expression rose in the hepatocytes of the liver for aged mice, whereas *ATG3* and *ATG7* expression was reduced [41]. The trend of *atg3* down-regulation in *A. japonicus* may be related to the inhibition of autophagic flux by *BMP9*.

## 5. Conclusions

In this study, we used MethylRAD-Seq to analyze the DNA methylation of body wall tissue of *A. japonicus* at different ages. DNA methylation profiles of body wall tissue of *A. japonicus* at different ages were constructed. Aspartate aminotransferase, succinate semialdehyde dehydrogenase, isocitrate dehydrogenase, *H2AX*, *Hsp90*, *Pepn*, *CDC6*, *Rasgap*, *slit1*, *ILK*, *Mtor*, *Pka*, and *atg3* were screened out as differentially methylated genes associated with age and aging. Therefore, we hypothesized that the DNA methylation levels and genetic patterns of *A. japonicus* changed during the developmental process. Future methylation analyses of gonads, respiratory trees, longitudinal muscles, and other tissues of *A. japonicus* will be conducted to provide a theoretical basis for the age judgment of *A. japonicus*.

## Figures and Tables

**Table 1:** Enzyme digestion reaction system and conditions.

Ingredient	Volume (µL/Single Sample; Digestion Group)	Volume (µL/Single Sample; Control Group)
DNA (1–200 ng/μL)	1	1
10× cut smart buffer	1.5	1.5
30× Enzyme	0.5	0.5
activator		
FspEI (5 U/μL)	0.8	0
Pure water	11.2	12
Total	15	15

**Table 2:** Connection reaction system and conditions.

Ingredient	Volume (µL/Single Sample)
enzyme digestion product	10
10× T4 ligase buffer	1
10 m M ATP	1
Adaptor 1 (5 µM)	0.8
Adaptor 2 (5 µM)	0.8
T4 DNA ligase (400 U/µL)	2
Pure water	5.4
Total	20

**Table 3:** PCR reaction system and conditions.

Ingredient	Volume (µL/Single Sample)	Reaction Conditions
Linked product	7	98 °C, 5 s
5× HF buffer	4	60 °C, 20 s
10 Mm dNTP	0.6	72 °C, 10 s
Primer 1 (10 µM)	0.4	20 cycles
Primer 2 (10 µM)	0.4	
Phusion high-fidelity DNA polymerase (2 U/µL)	0.2	
Pure water	7.4	
Total	20	

**Table 4:** Add barcode sequence PCR reaction system and conditions.

Ingredient	Volume (µL)	Reaction Conditions
Linked product	6	98 °C, 5 s
5× HF buffer	4	60 °C, 20 s
10 Mm dNTP	0.6	72 °C, 10 s
10 μM Primer3	0.2	6 cycles
10 μM Index Primer	0.2	
Phusion high-fidelity DNA polymerase (2 U/µL)	0.2	
Pure water	8.8	
Total	20	

**Table 5:** Adaptors and primers used for MethylRAD library preparation.

Adaptors and Primers	Sequence (5′ to 3′)
Adap-1 sense	ACACTCTTTCCCTACACGACGCTCTTCCGATCT
Adap-1 antisense	NNNNAGATCGGAAGAGC(AminoC6)
Adap-2 sense	GTGACTGGAGTTCAGACGTGTGCTCTTCCGATCT
Adap-2 antisense	NNNNAGATCGGAAGAGC(AminoC6)
Primers	
Primer1	ACACTCTTTCCCTACACGACGCT
Primer2	GTGACTGGAGTTCAGACGTGTGCT
Primer3	AATGATACGGCGACCACCGAGATCTACACTCTTTCCCTACACGACGCT
Index primer	CAAGCAGAAGACGGCATACGAGATXXXXXXGTGACTGGAGTTCAGACGTGT

**Table 6:** Statistical table of data volume change.

Sample	Raw_Reads	Clean_Reads	Percent
A1	27,876,786	13,004,227	64.47%
A2	25,394,643	9,778,024	48.56%
A3	22,576,882	13,989,100	68.32%
B1	21,983,545	11,520,479	56.76%
B2	20,331,587	11,673,620	58.19%
B3	20,264,854	9,132,031	45.60%
C1	22,270,807	9,663,839	47.27%
C2	21,435,672	10,214,922	50.25%
C3	21,629,651	10,513,567	51.96%
D1	23,925,401	10,439,003	52.18%
D2	26,942,187	10,401,084	51.91%
D3	25,788,921	9,946,640	49.54%

Note: 3-year-old (A), 2-year-old (B), 1-year-old (C), 4-month-old (D).

**Table 7:** Statistics of sequencing data of MethylRAD library.

Sample	Raw_Reads (Total)	Raw_Reads (Average)	Clean_Reads	Percent
A	75,848,311	25,282,770.33	36,771,351	48.48%
B	62,579,986	20,859,995.33	32,326,130	51.66%
C	65,336,130	21,778,710	30,392,328	46.52%
D	76,656,509	25,552,169.67	30,786,727	40.16%

Note: 3-year-old (A), 2-year-old (B), 1-year-old (C), 4-month-old (D).

**Table 8:** Methylation checkpoint coverage depth statistics.

Sample	CG_Site_Num	CWG_Site_Num
A1	62,148	5868
A2	79,810	10,589
A3	69,114	6808
Group A average	70,357	7755
B1	80,403	10,485
B2	77,861	8883
B3	82,863	14,770
Group B average	80,376	11,379
C1	81,634	14,770
C2	78,374	10,108
C3	77,188	9695
Group C average	79,065	11,524
D1	85,283	12,193
D2	82,424	11,537
D3	78,686	11,769
Group D average	82,131	11,833

Note: 3-year-old (A), 2-year-old (B), 1-year-old (C), 4-month-old (D).

## Data Availability

All of the data generated or analyzed during this study are included in this published article.

## Supplementary Materials

The following supporting information can be downloaded at: https://www.mdpi.com/article/10.3390/ani13223530/s1, Supplementary Document S1: Related Differentially Methylated Gene Annotation Information.

## References

[1] Liao Y.L. (1997). Zoology of China: Echinoderma: A. japonicuss.

[2] Fisheries Administration of the Ministry of Agriculture and Rural Affairs, National Fisheries Technology Promotion Station, Chinese Fisheries Society (2022). 2022 China Fisheries Statistical Yearbook.

[3] Zhang L.H., Ding J., Han Z.H., Chang Y.Q., Song J., Tian Y., Bai X.Q., Ding W.J. (2015). Study on the species and morphology of imitation A. japonicus bone fragments. J. Mar. Sci..

[4] Wang J.J., Liao M.J., Wang Y.G., Li B., Rong X.J., Ge J.L., Liu Q.B., Fan R.Y. (2023). Study on the variation law of the species and structure of imitation *A. japonicus* bone fragments with age. J. Sci. Fish Farm..

[5] Venney C.J., Johansson M.L., Heath D.D. (2016). Inbreeding effects on gene-specific DNA methylation among tissues of Chinook salmon. J. Mol. Ecol..

[6] Anastasiadi D., Piferrer F. (2020). A clockwork fish: Age prediction using DNA methylation-based biomarkers in the European seabass. J. Mol. Ecol. Resour..

[7] Zhang L., Jia F., Zhang G.L., Zeng L., Yi Y.J., Wang J.S. (2012). Research progress of plant DNA methylation. J. Anhui Agric. Sci..

[8] Lin Z.K., Xie F., Luo J.Y., Chen T., Xi Q.Y., Zhang Y.L., Sun J.J. (2023). Whole genome—Wide DNA methylation of longissimus dorsi muscle in Lantang and Landrace pigs. J. Northwest A F Univ. (Nat. Sci. Ed.).

[9] Wei S., Xie L.L., Zhu H., Zhang Q.J., Shen Y.B., Xu X.Y., Li J.L. (2023). Differential methylation analysis of Asian grass carp populations. Chin. J. Fish..

[10] Wang C.S., Huang X.D., Cui X.Y., Ni P., Ye S.G., Wang H., Gao D.X., Lei W. (2022). Effects of Vibrio harvei infection on DNA methylation of IL-6 gene of red-fin pufferfish. J. Dalian Ocean. Univ..

[11] Zhang Y.L., Zhou C.J. (2021). DNA methylation and fish age. J. Henan Fish..

[12] Mcgaughey D.M., Abaan H.O., Miller R.M., Kropp P.A., Brody L.C. (2014). Genomics of cpg methylation in developing and developed zebrafish. G3 Genes Genomes Genet..

[13] Montesanto A.D., Aquila P., Lagani V., Paparazzo E., Passarino G. (2020). A New Robust Epigenetic Model for Forensic Age Prediction. J. Forensic Sci..

[14] De paoli-iseppi R., Deagle B.E., Polanowski A.M., McMahon C.R., Dickinson J.L., Hindell M.A., Jarman S.N. (2019). Age estimation in a long-lived seabird (Ardenna tenuirostris) using DNA methylation-based biomarkers. Nat. Rev. Cancer.

[15] Cingolani P., Platts A., Wang L.L., Coon M., Nguyen T., Wang L., Land S.J., Lu X., Ruden D.M. (2012). A program for annotating and predicting the effects of single nucleotide polymorphisms, SnpEff: SNPs in the genome of Drosophila melanogaster strain w1118; iso-2; iso-3. Fly.

[16] Quinlan A.R., Hall I.M. (2010). BEDTools: A flexible suite of utilities for comparing genomic features. Bioinformatics.

[17] Sharma R., Patnaik S.K. (1987). Regulation of aspartate aminotransferase isoenzymes by hydrocortisone in the liver of aging rats. Arch. Gerontol. Geriatr..

[18] Ni J.J., Wu Z., Stoka V., Meng J., Hayashi Y., Peters C., Qing H., Turk V., Nakanishi H. (2019). Increased expression and altered subcellular distribution of cathepsin B in microglia induce cognitive impairment through oxidative stress and inflammatory response in mice. Aging Cell.

[19] Meng J., Liu Y.C., Xie Z., Qing H., Lei P., Ni J.J. (2020). Nucleus distribution of cathepsin B in senescent microglia promotes brain aging through degradation of sirtuins. Neurobiol. Aging.

[20] Sun Q.Y., Zhang C.Z., Mei B., Luo X., Zhu Z.M., Hua T.M. (2005). Age-related retinal γ-aminobutyric acid and neurofilament protein expression in cats. J. Anat..

[21] Shu J.B., Jiang S.Z., Meng Y.T. (2014). Research progress of succinic semialdehyde dehydrogenase deficiency. Contin. Med. Educ..

[22] Al-Zghoul M.B., Ismail Z.B., Dalab A.S., Al-Ramadan A., Althnaian T.A., Al-ramadan S.Y., Ali A.M., Albokhadaim I.F., Al Busadah K.A., Eljarah A. (2015). Hsp90, Hsp60 and HSF-1 genes expression in muscle, heart and brain of thermally manipulated broiler chicken. Res. Vet. Sci..

[23] Bansal G.S., Norton P.M., Latchman D.S. (1991). The 90-kDa heat shock protein protects mammalian cells from thermal stress but not from viral infection. Exp. Cell Res..

[24] Boehm A.K., Seth M., Mayr K.G., Fortier L.A. (2007). Hsp90 mediates insulin-like growth factor 1 and interleukin-1beta signaling in an age-dependent manner in equine articular chondrocytes. Arthr. Rheum..

[25] Johanna S.S., Bernard R. (2016). Effects of Aging and Oxidative Stress on Spermatozoa of Superoxide-Dismutase 1- and Catalase-Null Mice1. Biol. Reprod..

[26] Miska K.B., Fetterer R.H., Wong E.A. (2014). The mRNA expression of amino acid transporters, aminopeptidase N, and the di- and tri-peptide transporter PepT1 in the embryo of the domesticated chicken (Gallus gallus) shows developmental regulation. Poult. Sci..

[27] Singh Yadav R.N., Singh S.N. (1980). Regulation of NAD- and NADP-linked isocitrate dehydrogenase by hydrocortisone in the brain and liver of male rats of various ages. Biochim. Biophys. Acta (BBA)-Gen. Subj..

[28] Markopoulos G.S., Roupakia E., Tokamani M., Vartholomatos G., Tzavaras T., Hatziapostolou M., Fackelmayer F.O., Sandaltzopoulos R., Polytarchou C., Kolettas E. (2017). Senescence-associated microRNAs target cell cycle regulatory genes in normal human lung fibroblasts. Exp. Gerontol..

[29] Pankiewicz A., Sledzinski T., Nogalska A., Swierczynski J. (2003). Tissue specific, sex and age—Related differences in the 6-phosphogluconate dehydrogenase gene expression. Int. J. Biochem. Cell Biol..

[30] Kim J.H., Lee H.K., Takamiya K., Huganir R.L. (2003). The role of synaptic GTPase-activating protein in neuronal development and synaptic plasticity. J. Soc. Neurosci..

[31] Saito S., Kawamura T., Higuchi M., Kobayashi T., Yoshita-Takahashi M., Yamazaki M., Abe M., Sakimura K., Kanda Y., Kawamura H. (2015). RASAL3, a novel hematopoietic RasGAP protein, regulates the number and functions of NKT cells. Eur. J. Immunol..

[32] Srivastava K., Tripathi R., Mishra R. (2018). Age-dependent alterations in expression and co-localization of Pax6 and Ras-GAP in brain of aging mice. J. Chem. Neuroanat..

[33] Ethell I.M., Irie F., Kalo M.S., Couchman J.R., Pasquale E.B., Yamaguchi Y. (2001). EphB/syndecan-2 signaling in dendritic spine morphogenesis. Neuron.

[34] Morita A., Yamashita N., Sasaki Y., Uchida Y., Nakajima O., Nakamura F., Yagi T., Taniguchi M., Usui H., Katoh-Semba R. (2006). Regulation of dendritic branching and spine maturation by semaphorin3A-Fyn signaling. J. Soc. Neurosci..

[35] Yoshida J., Kubo T., Yamashita T. (2008). Inhibition of branching and spine maturation by repulsive guidance molecule in cultured cortical neurons. Biochem. Biophys. Res. Commun..

[36] El-Hoss J., Arabian A., Dedhar S., St-Arnaud R. (2014). Inactivation of the integrin-linked kinase (ILK) in osteoblasts increases mineralization. Gene.

[37] Weichhart T. (2018). mTOR as Regulator of Lifespan, Aging, and Cellular Senescence: A Mini-Review. Gerontology.

[38] Erdogan C.S., Hansen B.W., Vang O. (2016). Are invertebrates relevant models in ageing research? Focus on the effects of rapamycin on TOR. Mech. Ageing Dev..

[39] Zacharewicz E., Della Gatta P., Reynolds J., Garnham A., Crowley T., Russell A.P., Lamon S. (2014). Identification of microRNAs linked to regulators of muscle protein synthesis and regeneration in young and old skeletal muscle. PLoS ONE.

[40] Li N., Shi R., Ye Y., Zhang Y., Zhang Y., Wang Z., Gu Y., Yin Y., Chen D., Tang J. (2022). Aging-Induced Down-Regulation of PKA/BKCa Pathway in Rat Cerebral Arteries. Physiol. Res..

[41] Liu R., Xu W., Zhu H., Dong Z., Dong H., Yin S. (2023). Aging aggravates acetaminophen-induced acute liver injury and inflammation through inordinate C/EBPα-BMP9 crosstalk. Cell Biosci..

